# Current and Future Potential Impact of COVID-19 on Kratom (*Mitragyna speciosa* Korth.) Supply and Use

**DOI:** 10.3389/fpsyt.2020.574483

**Published:** 2020-11-26

**Authors:** Darshan Singh, Paula N. Brown, Eduardo Cinosi, Ornella Corazza, Jack E. Henningfield, Albert Garcia-Romeu, Christopher R. McCurdy, Lance R. McMahon, Walter C. Prozialeck, Kirsten E. Smith, Marc T. Swogger, Charles Veltri, Zach Walsh, Oliver Grundmann

**Affiliations:** ^1^Centre for Drug Research, University Sains Malaysia, George Town, Malaysia; ^2^Centre for Applied Research and Innovation, British Columbia Institute of Technology, Burnaby, BC, Canada; ^3^Department of Clinical and Pharmaceutical Sciences, School of Life and Medical Sciences, University of Hertfordshire, Hatfield, United Kingdom; ^4^Hertfordshire Partnership National Health Service University Foundation Trust, St Albans, United Kingdom; ^5^Department of Psychiatry and Behavioral Sciences, Johns Hopkins University School of Medicine, Baltimore, MD, United States; ^6^Pinney Associates, Bethesda, MD, United States; ^7^Department of Psychiatry and Behavioral Sciences, Johns Hopkins University School of Medicine, Baltimore, MD, United States; ^8^Department of Medicinal Chemistry, College of Pharmacy, University of Florida, Florida, FL, United States; ^9^Department of Pharmacodynamics, College of Pharmacy, University of Florida, Florida, FL, United States; ^10^Department of Pharmacology, College of Graduate Studies, Midwestern University, Downers Grove, IL, United States; ^11^National Institute on Drug Abuse Intramural Research Program, Baltimore, MD, United States; ^12^Department of Psychiatry, University of Rochester Medical Center, Rochester, NY, United States; ^13^Department of Pharmaceutical Sciences, College of Pharmacy, Midwestern University, Glendale, AZ, United States; ^14^Department of Psychology, University of British Columbia, Kelowna, BC, Canada

**Keywords:** COVID-19, kratom, SUD, OUD, withdrawal

## Abstract

Kratom (*Mitragyna speciosa* Korth., Rubiaceae) is native to and has traditional use in Southeast Asia. The number of kratom users outside of Southeast Asia has increased significantly in recent decades with use spreading to the Unites States (US) and Europe. Because of its reputed opioid-like psychoactive effects at higher doses, kratom has been regulated in several countries and is subject to an import ban by the US Food and Drug Administration. Nonetheless, in the US it is estimated that 10–15 million people consume kratom primarily for the self-treatment of pain, psychiatric disorders, to mitigate withdrawal from or dependence on opioids, and to self-treat opioid use disorder or other substance use disorders (SUDs). Due to the global COVID-19 pandemic, a shortage in the supply of kratom products may place unexpected burdens on kratom users, potentially influencing some who use kratom for SUD self-treatment to regress to harmful drug use, hence increasing the likelihood of adverse outcomes, including overdose. Inadequate treatment, treatment barriers, and increases in the sales of adulterated kratom products on the internet or in convenience stores could exacerbate circumstances further. Although there are currently no verified indications of kratom scarcity, researchers and clinicians should be aware of and remain vigilant to this unanticipated possibility.

## Introduction

Kratom (*Mitragyna speciosa* Korth., Rubiaceae) is a tree native to Southeast Asia with psychoactive properties due to the presence of indole alkaloids (1, 2). The primary alkaloid, mitragynine, has been shown to interact with μ-opioid receptors as a biased partial agonist leading to analgesia (3). In addition, kratom products may also produce dose- and strain-dependent stimulant and sedative effects (4). Chronic consumption at high doses has a potential to cause dependence and withdrawal symptoms (5) consistent with a Substance Use Disorder (SUD) (6, 7). A majority of user surveys and numerous observational studies suggest that kratom is widely used in Western nations for a range of conditions, including self-treatment of acute and chronic pain, psychiatric conditions, such as depressive and anxiety disorders, and mitigation of withdrawal symptoms from addictive drugs, both illicit and prescribed, particularly opioid-based medications (8). Among polydrug users and those with a history of SUD, kratom has also been consumed as a means of reducing use of or abstaining from dangerous prescription opioids and heroin (9, 10). Adverse effects of kratom use have been reported in several cases of polydrug use with opioids, benzodiazepines, and acetaminophen primarily resulting in seizures, hepatotoxicity, and gastrointestinal symptoms (11). Polydrug exposure involving kratom increases the odds ratio of more serious adverse events occurring, including admittance to a healthcare facility and occurrence of more serious medical outcomes such as hepatic damage and death (12, 13). Kratom withdrawal symptoms are similar to those of opioids but with lower severity, presenting with transient gastrointestinal upset, muscle and nerve pain, insomnia, sweating, tremor, fatigue, and psychological distress including restlessness, irritability, increased cravings, depressed mood, and anxiety. Buprenorphine in combination with clonidine may prove to be a clinically effective treatment for most of these symptoms as indicated by case reports, although these drugs are associated with their own adverse effects (5). However, in traditional settings, kratom users have their own methods for mitigating kratom withdrawal symptoms.

The widespread use of kratom and consistent reports of its benefits or therapeutic value that are important to users raises the question: would sudden decreases in the availability of the plant have negative impacts on kratom users? Various internet studies found that some kratom users are concerned about the possibility of relapsing to opioids and/or seeking alternative, possibly questionable, sources of kratom if products become less readily available. This is a serious concern as kratom, not currently regulated as a dietary supplement, may be adulterated by unscrupulous traders and cause users to relapse to opioid use and inevitably experience a significant increase in overdose risk (7, 9, 14–17). Indeed, there is evidence to suggest that the COVID-19 pandemic has been associated with increased drug overdose deaths and that the reduced access to conventional treatment, as well as mutual-aid groups, is a plausible contributing factor (18), though it is unknown whether diminished access to kratom has explicitly contributed to any overdose deaths.

### Possible Kratom Scarcity and Misuse in the Context of COVID-19

Because of the potential public health impact of kratom scarcity and the international implications of COVID-19, the probable impact of the global pandemic on kratom availability is of significant interest in regard to consumption patterns. Specifically, COVID-19-related disruptions in kratom access/supply and use could increase the likelihood that users turn to more readily available, but more dangerous, products to self-treat symptoms they had primarily used kratom for. Even prior to the pandemic, the kratom supply chain experienced significant, repeated disruptions and episodes of consumer uncertainty. This was at least partially due to the import alert issued by FDA in February 2014 which resulted in companies restricting inventory to avoid FDA seizure (19). Another concern that COVID-19 raises in addition to potential supply chain disruptions is the possibility that people may use or misuse kratom in an attempt to inoculate themselves from COVID-19 infection or to self-treat the various symptoms associated with COVID-19, despite no scientific support for kratom use in such a manner (20, 21).

### Origin of Anecdotal Accounts

Although the obtained information is anecdotal, we were able to solicit informal accounts from kratom growers in Malaysia and vendors in the United States (Arizona, Florida, and Illinois) and Europe. Kratom users also provided us with information on the state of kratom supply and personal consumption. Due to the fast-moving nature of COVID-19, we relied on informal, personal networks and publicly advertised vendors to compile a sense of the situation over a period of 3 months between March and May 2020, rather than undertake a systematic study of a continually evolving situation. We believe that these anecdotal accounts will help researchers identify key areas of focus in the coming year.

## Discussion

### Kratom Growers and Vendors

Using community and personal contacts, we were alerted to several important factors that warrant investigation. First, due to shelter-in-place orders and social distancing restrictions, kratom growers in Malaysia experienced problems selling their harvest. Further, the initial rigid phase of the movement control order disrupted distribution of kratom supply from kratom plantations to consumers, chiefly among those who have been using kratom to self-treat SUDs. Disrupted trade routes via sea or air have been reported for some kratom products, although it is unknown to what degree this has impacted global kratom supply to date. Kratom vendors in the US and Europe, despite the imposed import bans, primarily obtain their kratom supplies from Indonesia which is the main global exporter for kratom (22). The majority of vendors have not seen changes in supplies of kratom products since December 2019 although they expressed uncertainty as to whether that may change in the future if COVID-19 leads to the imposition of additional commercial restrictions. In response to the uncertainty of the kratom market, many vendors have increased their stock supply in recent months in preparation for potential pandemic-related disruptions. Distributors who obtain a majority of their kratom product from Thailand were able to continue typical purchasing levels until February 2020, which resulted in a stockpile due to shop closures related to mandated lock-downs and social distancing. Vendors are expecting a resumption in purchasing now that some shops are reopening, and anticipate a return to typical sales volume. Though kratom vendors noticed an increased demand for kratom products among users in recent months, they did not perceive that the increase was associated with a novel indication or different uses of kratom. At least one vendor associated the increased demand with the Netflix production “A Leaf of Faith” (released 2018 but still available on Netflix), having several new customers mention their decision to try kratom as a result of having watched the documentary, rather than COVID-19 related issues.

### Kratom Users

Given disruptions described above, kratom users in Malaysia encountered problems obtaining their regular kratom supply. The problem worsened when enforcement agencies raided illegal kratom ports in the community—making it more difficult for opioid users and people with SUDs who were self-treating their dependence with kratom to obtain their regular supply. Similarly, due to COVID-19, manual laborers who were daily wage earners lost their income and could not afford kratom products to self-treat medical conditions. Most US kratom users did not discuss difficulties with obtaining kratom products from their usual sources since the outbreak of COVID-19 in their respective locality. Still, users were cognizant of the possibility of kratom shortages if the pandemic continues. Many users feared that they may not have access to their usual products for the rest of the year. To date, this fear has not resulted in users stockpiling kratom, likely due to limited affordability (e.g., most people could not afford to hoard kratom like other, less expensive commodities). Few users mentioned increasing their kratom consumption during COVID-19. Reasons for use primarily centered on alleviating stress or psychiatric disorder symptoms (e.g., anxiety and depression), or continuation of kratom as a means of addressing SUD symptoms. Given the limited number of kratom users informally consulted (*n* = 42), these anecdotes cannot be generalized. Of concern, some sources have noted increases in unscientific claims made by irresponsible vendors regarding kratom's supposed “anti-coronavirus” properties (23). The FDA is issuing warnings to such disreputable vendors and kratom advocacy organizations are condemning misinformation through consumer advisory postings, though the degree to which this misinformation is spreading to users remains unclear and if it differs by nation (21, 23).

### Potential Implications of COVID-19 on Kratom Availability and Use

Our on-the-ground conversations provided an outlook of how kratom growers, vendors, and users perceive COVID-19 and its impact, providing a starting point for systematic investigation. According to published user surveys, common reasons for kratom use include the self-treatment of acute and chronic pain, psychological distress, mitigation of dependence and/or withdrawal symptoms from an illicit or prescription drug use (7, 9, 15). While the ongoing pandemic has created uncertainty among vendors and users about kratom availability, it has not, to date, impacted the actual availability of the product in the US. Considering the potential importance of kratom as a self-treatment strategy or harm-reduction component for SUDs, an unanticipated supply disruption may lead to a rise in opioid and other drug use with subsequently increased risk for overdose and fatality. Reduced kratom access may also negatively impact the well-being of individuals who use kratom for the acute relief of psychological distress at a time of increasing socioeconomic uncertainty and stress. COVID-19-related disruptions in kratom availability may also influence or coerce regular users to try more harmful herbal, synthetic, or plant-based New Psychoactive Substances or even illicit drugs in self-managing their aggravating health conditions (24). In an unexpected situation, if there is an imminent increase in kratom fatalities/toxicities arising from the COVID-19 pandemic, enforcement agencies may use the scenario as a precedent to legally or effectively ban kratom use.

Based on import and sales of kratom, there are an estimated 10–15 million kratom users in the US, meaning that disruptions for even a small proportion of regular users could result in an outsized effect (25). In the coming months, it will be important to monitor kratom supplies and purchasing avenues (e.g., Internet and local shops). The Internet will likely be an increasing method for monitoring sales, user reaction to COVID-19, issues related to supply, and motivations for use during the pandemic. It will also be important to raise awareness among healthcare professionals if current kratom users circumstantially experience shortages. In such cases, regular kratom users may come to the clinical attention of healthcare professionals, possibly requiring prescribed treatment options in the absence of kratom (e.g., anxiolytics, antidepressants, analgesics, and opioid agonist therapies). Further information is also needed to improve our understanding on how the impact of COVID-19 is affecting kratom users in terms of obtaining unadulterated kratom products, as well as other important occurrences that could affect kratom supply, patterns of use and its therapeutic popularity among users.

## Data Availability Statement

The data generated in this study is subject to the following licenses/restrictions: Datasets are on a secured cloud drive as part of larger datasets. Requests to access these datasets should be directed to Oliver Grundmann, grundman@ufl.edu.

## Author Contributions

DS and OG conceptualized the paper. All authors revised and finalized the paper.

## Conflict of Interest

JH is an employee of Pinney Associates which provides consulting services on the development and regulation of pharmaceuticals, cannabinoids, and dietary supplements including kratom and advises the American Kratom Association on Kratom science. PB provides scientific guidance on dietary supplement manufacture and regulatory compliance to companies, associations, and government and in her capacity as Director of Applied Research at BCIT engages in contract research on medicinal plants, including Kratom. AG-R serves as a scientific advisor for ETHA Natural Botanicals, a distributor of kratom supplements. The remaining authors declare that the research was conducted in the absence of any commercial or financial relationships that could be construed as a potential conflict of interest. The handling editor and two of the reviewers, JC and SC, declared a shared affiliation, though no collaboration, with two of the authors, EC and OC, at the time of review.
